# Infiltration of hidden antimicrobial resistance among healthy people in a Japanese community

**DOI:** 10.1093/jacamr/dlac031

**Published:** 2022-03-25

**Authors:** Akira Fukuda, Hiromi Nakamura, Kaoru Umeda, Kaori Yamamoto, Yuji Hirai, Masaru Usui, Jun Ogasawara

**Affiliations:** Food Microbiology and Food Safety Unit, Division of Preventive Veterinary Medicine, School of Veterinary Medicine, Rakuno Gakuen University, Ebetsu, Japan; Division of Microbiology, Osaka Institute of Public Health, Osaka, Japan; Division of Microbiology, Osaka Institute of Public Health, Osaka, Japan; Division of Microbiology, Osaka Institute of Public Health, Osaka, Japan; Division of Microbiology, Osaka Institute of Public Health, Osaka, Japan; Food Microbiology and Food Safety Unit, Division of Preventive Veterinary Medicine, School of Veterinary Medicine, Rakuno Gakuen University, Ebetsu, Japan; Division of Microbiology, Osaka Institute of Public Health, Osaka, Japan

## Abstract

**Background:**

Under non-antimicrobial selective pressure, antimicrobial-resistant bacteria do not easily become dominant in the microbiota. Furthermore, their low levels prevent detection by isolation, resulting in an underestimation of the prevalence of antimicrobial-resistant bacteria.

**Objectives:**

We evaluated the infiltration of antimicrobial-resistant bacteria and their related β-lactamase genes among healthy people in non-clinical settings.

**Methods:**

Cephalosporin- and fluoroquinolone-resistant *Escherichia coli* and *bla* genes were quantified in 217 faecal samples from healthy people in non-clinical settings in Japan. *E. coli* colonies grown on deoxycholate hydrogen sulphide-lactose (DHL) agar, with and without antimicrobials (cefotaxime and ciprofloxacin), were quantified, and *E. coli* isolates were analysed for their susceptibility to antimicrobials and the presence of *bla* genes. DNA extracted from faecal samples was used to quantify *bla* genes using quantitative PCR (qPCR).

**Results:**

The isolation rates of cefotaxime- and ciprofloxacin-resistant *E. coli* were 6.9% and 12.4%, respectively, using agars without antimicrobials, and 12.0% and 24.4%, respectively, using agars with antimicrobials. For samples from which cefotaxime- and ciprofloxacin-resistant *E. coli* were isolated only using agars with antimicrobials, the ratios of cfu on DHL agars with and without antimicrobials were below −2 log. *E. coli* harbouring *bla* genes were isolated from 35.0% of the faecal samples using agars, and *bla* genes were detected in 65.0% of faecal DNA samples using qPCR.

**Conclusions:**

Among people carrying cefotaxime- and ciprofloxacin-resistant *E. coli* in non-clinical settings, cefotaxime- and ciprofloxacin-resistant *E. coli* were not dominant in half of the subjects. These individuals may play a role as reservoirs of antimicrobial-resistant bacteria.

## Introduction

The spread of antimicrobial resistance (AMR) is one of the major global concerns, with outflow from clinical settings to non-clinical settings being an important transmission route.^1,2^ Non-clinical settings may act as reservoirs of AMR.^3,4^ In human communities, AMR transmission from non-clinical settings to clinical settings could be a risk factor for nosocomial infections.

Antimicrobial-resistant bacteria (ARB) and antimicrobial resistance genes (ARGs) show survival disadvantages under non-antimicrobial selective pressure, as there is a fitness cost to maintain AMR.^5^ Specific clones of disseminated ARB and plasmids carrying ARGs show greater or equal growth abilities as that of wild-type clones, regardless of the presence of antimicrobial selective pressure.^6,7^  *Escherichia coli* ST131 clones, which are frequently isolated from patients in clinical settings, form a pandemic clonal group that is antimicrobial-resistant, particularly to fluoroquinolone and cephalosporin.^8^ ESBL genes have been detected in various sources; in Osaka, the specific plasmid harbouring *bla*_IMP-6_ continues to persist in clinical settings.^9,10^ Dissemination of these clones and plasmids may contribute to the spread and maintenance of AMR among the bacteria that much easier.

The prevalence of antimicrobial-resistant *E. coli* among people is lower in non-clinical than clinical settings,^4^ as exposure to antimicrobials is lower; thus, ARB do not typically dominate the microbiota.^5^ In a surveillance, dominant bacteria were readily isolated, and ARB prevalence was evaluated on only these bacteria. Selective agars, such as those containing antimicrobials, improved the detection rates of ARB among the major populations of susceptible bacteria.^11^ Therefore, the use of appropriate techniques to detect ARB is important for understanding the infiltration of these bacteria in non-clinical settings.

Here, we aimed to evaluate the infiltration of ARB and ARGs among healthy people from Japan in non-clinical settings. We quantified cephalosporin-, fluoroquinolone- and carbapenem-resistant *E. coli* and ARGs, and evaluated the prevalence of minority ARB and ARGs.

## Materials and methods

The study was approved by the Ethical Review Committee of the Osaka Institute of Public Health (approval no. 1808-05-3).

Faecal samples (*n *= 217) were collected from healthy people in non-clinical settings in Osaka, Japan, in 2019. To determine the concentration of *E. coli* (cfu/g), faecal samples (0.2 g) were homogenized in 1 mL sterile saline and serially diluted and plated on deoxycholate hydrogen sulphide-lactose (DHL) agar without antimicrobials (DHL-O) or with the following antimicrobials: 2 mg/L cefotaxime (DHL-C), 0.5 mg/L ciprofloxacin (DHL-Q), or 0.125 mg/L meropenem (DHL-M).^12,13^ After incubation, *E. coli* colonies were counted according to their colony morphology, and one colony was isolated from each agar. Additionally, to isolate *E. coli* strains from faecal samples that were not isolated from DHL agars with antimicrobials during the previous procedure, the faecal samples (0.2 g) were inoculated into 15 mL EC broth and incubated at 37°C for 24 h. The preculture broth was streaked onto agars and incubated. One colony was isolated from each agar. Strains were identified as *E. coli* by specific primers.^4^

Susceptibility of the strains to ampicillin, cefazolin, cefotaxime, nalidixic acid, ciprofloxacin and meropenem was evaluated using the microbroth dilution method.^12^

The *bla*_CTX-M_, *bla*_TEM_, *bla*_SHV_ and AmpC β-lactamase genes were analysed using PCR, and subtypes of *bla*_CTX-M_ were identified via sequencing (Table S1, available as Supplementary data at *JAC-AMR* Online).^4^ ST131 clones were identified using PCR (Table S1).^8^

DNA was extracted from 0.2 g of faecal samples using an ISOFECAL Kit (Nippon Gene, Tokyo, Japan) and served as the template for quantitative PCR (qPCR) to determine the copy numbers of *bla*_CTX-M_, *bla*_TEM_, *bla*_SHV_, *bla*_IMP_, 16S rRNA and *uidA* using TB Green® Premix Ex Taq^™^ II (Table S1).^3,14,15^

Statistical significance was determined using the χ^2^ test to compare the isolation rates of ARB and the Mann–Whitney *U*-test to compare cfu and the ratios of cfu on DHL with and without antimicrobials for each of the faecal samples. The significance was set at *P < *0.05.

## Results

*E. coli* strains were isolated from 217, 26 and 53 faecal samples using DHL-O, DHL-C and DHL-Q, respectively. No colonies were observed using DHL-M. The isolation rates of antimicrobial-resistant *E. coli* from healthy individuals using DHL agars without and with antimicrobials are shown in Table 1. In strains isolated from healthy people in Japan using agar without antimicrobials (DHL-O), the resistance rates for ampicillin, cefazolin and nalidixic acid were approximately 30% each, and those for cefotaxime and ciprofloxacin were 6.9% and 12.4%, respectively. All strains isolated using DHL-C and DHL-Q showed resistance to cefotaxime and ciprofloxacin, respectively. No strains were resistant to meropenem. Additionally, using DHL-C and DHL-Q, 11 cefotaxime- and 26 ciprofloxacin-resistant *E. coli* strains, respectively, were isolated from faecal samples from which susceptible strains were isolated using DHL-O. The isolation rates from faecal samples using agars without and with antimicrobials for cefotaxime- and ciprofloxacin-resistant *E. coli* strains were 12.0% and 24.4%, respectively. The isolation rates of quinolone-resistant *E. coli* strains using a combination of DHL-O and DHL-Q was improved compared with that using only DHL-O (*P < *0.05). For cefotaxime-resistant *E. coli*, a combination of DHL-O and DHL-C tended to lead to higher isolation rates than using only DHL-O (*P = *0.07).

**Table 1.** Rates of isolation of antimicrobial-resistant *E. coli* strains from faecal samples (*n *= 217) using agars without or with antimicrobials

Isolation rates of resistant strain from faeces (*n *= 217)	Without antimicrobials	Combination of without and with antimicrobials		Total (without/with antimicrobials)
		cefotaxime	ciprofloxacin	
Ampicillin	32.7%	36.9%	38.2%	38.7%
	(71)	(80)	(83)	(84)
Cefazolin	29.0%	33.2%	34.1%	35.0%
	(63)	(72)	(74)	(76)
Cefotaxime	6.9%	12.0%**	10.1%	12.0%**
	(15)	(26)	(22)	(26)
Nalidixic acid	27.6%	30.4%	38.7%*	38.7%*
	(60)	(66)	(84)	(84)
Ciprofloxacin	12.4%	15.7%	24.4%*	24.4%*
	(27)	(34)	(53)	(53)

None of the strains was isolated from agars with meropenem. None of the strains exhibited resistance to meropenem.

* *P < *0.05 compared with the rate without antimicrobials.

** *P < *0.1 compared with the rate without antimicrobials.

**Figure 1.**
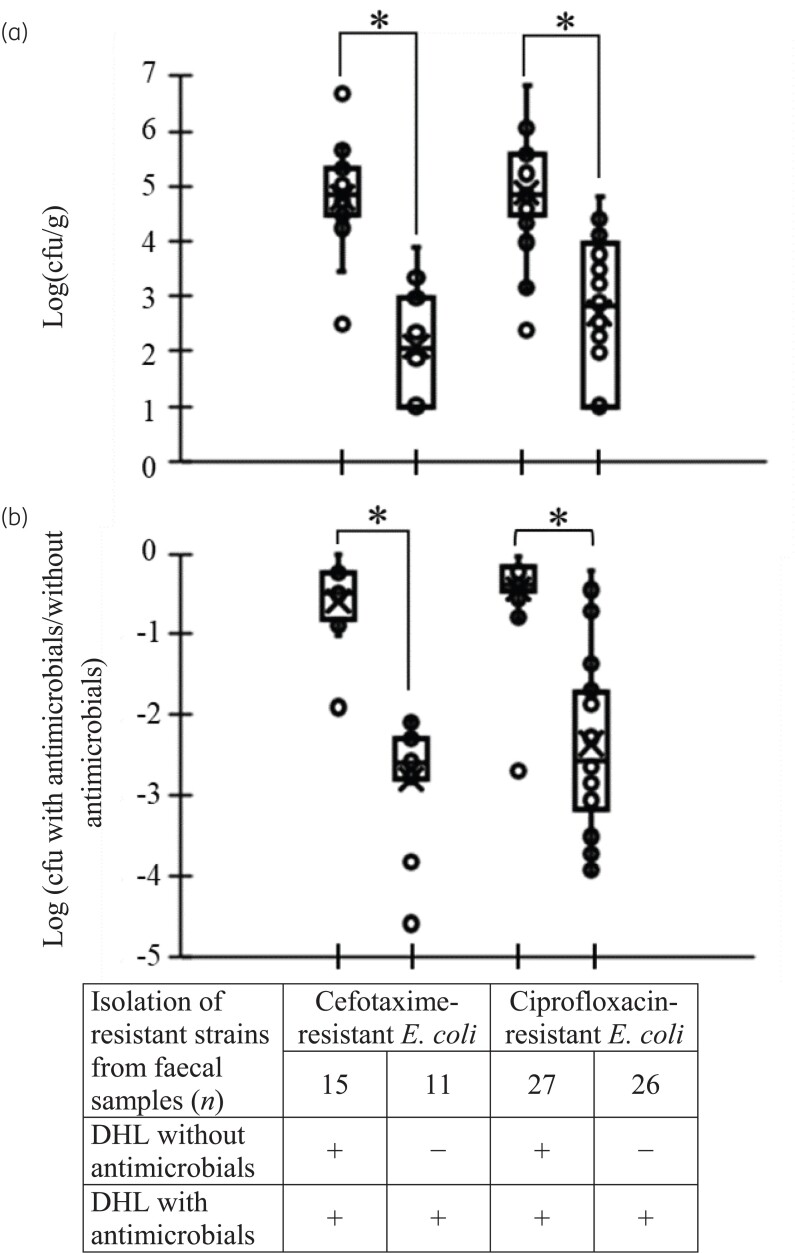
Load of antimicrobial-resistant bacteria in faecal samples. (a) Concentrations of antimicrobial-resistant *E. coli* by cfu on DHL agars supplemented with antimicrobials (cefotaxime and ciprofloxacin). (b) Comparison between ratios of cfu on DHL agars supplemented and not supplemented with antimicrobials (cefotaxime and ciprofloxacin). *P < 0.05.

On DHL-O, the mean number of faecal sample *E. coli* colonies was 4.7 ± 1.0 log cfu/g. The total cfu and the cfu ratios (with/without antimicrobials) for cefotaxime- and ciprofloxacin-resistant *E. coli* strains isolated sequentially from DHL without and then with the respective antimicrobial were higher than those isolated from strains only grown on DHL with antimicrobials (*P < *0.05) (Figure 1). For samples from which cefotaxime- and ciprofloxacin-resistant *E. coli* were isolated using an agar without antimicrobials, the cfu ratios were more than −1 log. For samples from which cefotaxime- and ciprofloxacin-resistant *E. coli* were isolated only using agars with antimicrobials, the cfu ratios were below −2 log.

*E. coli* harbouring *bla* genes were isolated from 76 (35.0%) faecal samples: *bla*_TEM_ (*n *= 59) showed the highest prevalence, followed by *bla*_CTX-M_ (*n *= 23), *bla*_CMY-2_ (*n *= 3) and *bla*_DHA_ (*n *= 1). The dominant subtypes of *bla*_CTX-M_ were *bla*_CTX-M-14_ (*n *= 9) and *bla*_CTX-M-27_ (*n *= 7) in the *bla*_CTX-M-9_ group, followed by *bla*_CTX-M-15_ (*n *= 5) and *bla*_CTX-M-55_ (*n *= 2) in the *bla*_CTX-M-1_ group (Table S2).

Using DHL-O and a combination of DHL without and with antimicrobials, ST131 clones were isolated from 22 (10.1%) and 27 (12.4%) faecal samples, respectively.

Using qPCR, *bla* genes were detected in 141 (65.0%) faecal samples; *bla*_TEM_, *bla*_CTX-M_ and *bla*_SHV_ were detected in 69 (31.8%), 33 (15.2%) and 99 (45.6%) DNA extracts from the faecal samples, respectively, whereas *bla*_IMP_ was not detected. In *bla* genes detected by qPCR, the mean copy numbers of *bla*_TEM_, *bla*_CTX-M_ and *bla*_SHV_ were 7.5 ± 1.7, 5.6 ± 1.5 and 3.3 ± 0.7 log copies/g, respectively. There were no significant differences in the mean copy number of *bla* genes and the ratios of *bla* to 16S rRNA or *uidA* genes between faecal samples containing *E. coli* strains harbouring the corresponding *bla* genes whether isolated or not.

## Discussion

The prevalence of resistance to cephalosporins, fluoroquinolones and carbapenems among people in non-clinical settings was lower than that in clinical settings,^4,9,16–18^ and some healthy people in non-clinical settings showed low concentrations and ratios of ARB with a dominance of susceptible bacteria. Moreover, the detection rates of cephalosporin- and fluoroquinolone-resistant bacteria were about 2-fold higher among healthy people in non-clinical settings when using agars with antimicrobials than without antimicrobials, revealing the true prevalence of ARB. Selective agars, such as those containing antimicrobials, enable the isolation of ARB more readily and sensitively. Some commercially available selective agars can be used; however, these screening methods are only available for limited types of bacteria and antimicrobials.^11^ In clinical settings, even in individuals with low ARB concentrations, ARB are selected by treatment of infectious disease using antimicrobials, making treatment difficult.^16^ Healthy people harbouring ARB serve as carriers and reservoirs of ARB and then, to simplify ARB isolation, more sensitive and specific detection methods are needed.

The *bla* genes were detected in 65.0% of faecal samples, including some that could not be isolated from *E. coli* strains harbouring *bla* genes. Among the *E. coli* strains, distribution patterns of *bla* genes were similar to those observed in southeast Asian countries, including in Japan.^8,19^ ARGs can be transferred among bacteria across genera via mobile genetic elements, such as plasmids.^20^ Commercial kits for detecting ARGs are available for use in clinical settings; these kits provide an easier and more rapid detection method for ARGs than bacterial isolation methods.^21^ The dissemination of ARGs is evaluated;^2,3^ however, the causative ARB and transmission routes are difficult to determine without isolating the ARB. By quantifying and detecting ARGs, hotspots can be revealed as dissemination indicators and effective treatment can be administered.^2^

In our study, *E. coli* ST131 clones were isolated from faecal samples of healthy people regardless of whether agars containing antimicrobials were used. In non-clinical settings, foods contaminated with ST131 clones may contribute to bacterial spread among healthy people.^22^ To control the dissemination of ARB among humans, it is necessary to comprehensively understand the transmission routes, circulation and persistence of ARB in non-clinical settings and the transmission of ARB from/to non-clinical settings and clinical settings.

In conclusion, half of the healthy people in non-clinical settings carried a minority of cephalosporin- and quinolone-resistant *E. coli* within their microbiota, and minority ARB are difficult to isolate without using selective agars. These people may act as reservoirs of ARB, leading to an underestimation of the risk for ARB dissemination in human communities. Moreover, the quantification of ARGs could provide supporting data on the prevalence and dissemination of AMR. In human communities, people move between non-clinical and clinical settings with contact occurring between these two groups. Hygiene management is important for controlling the dissemination of infectious disease including ARB.

## Supplementary Material

dlac031_Supplementary_Data

## Supplementary data

Table S1 and Table S2 are available as Supplementary data at *JAC-AMR* Online.
